# An algorithm for classifying tumors based on genomic aberrations and selecting representative tumor models

**DOI:** 10.1186/1755-8794-3-23

**Published:** 2010-06-22

**Authors:** Xin Lu, Ke Zhang, Charles Van Sant, John Coon, Dimitri Semizarov

**Affiliations:** 1 Global Pharmaceutical Research and Development, Abbott Laboratories, 100 Abbott Park Road, Building AP-10, Dep. R4CD, Abbott Park, IL 60064, USA; 2 Department of Pathology, School of Medicine & Health Sciences, University of North Dakota, 501 N. Columbia Road, Grand Forks, ND 58202, USA; 3 Astellas Pharma Global Development, INC. 8045 Lamon Ave, Skokie, IL 60077, USA; 4 Department of Pathology, Rush University Medical Center, Chicago, IL 60612, USA

## Abstract

**Background:**

Cancer is a heterogeneous disease caused by genomic aberrations and characterized by significant variability in clinical outcomes and response to therapies. Several subtypes of common cancers have been identified based on alterations of individual cancer genes, such as HER2, EGFR, and others. However, cancer is a complex disease driven by the interaction of multiple genes, so the copy number status of individual genes is not sufficient to define cancer subtypes and predict responses to treatments. A classification based on genome-wide copy number patterns would be better suited for this purpose.

**Method:**

To develop a more comprehensive cancer taxonomy based on genome-wide patterns of copy number abnormalities, we designed an unsupervised classification algorithm that identifies genomic subgroups of tumors. This algorithm is based on a modified genomic Non-negative Matrix Factorization (gNMF) algorithm and includes several additional components, namely a pilot hierarchical clustering procedure to determine the number of clusters, a multiple random initiation scheme, a new stop criterion for the core gNMF, as well as a 10-fold cross-validation stability test for quality assessment.

**Result:**

We applied our algorithm to identify genomic subgroups of three major cancer types: non-small cell lung carcinoma (NSCLC), colorectal cancer (CRC), and malignant melanoma. High-density SNP array datasets for patient tumors and established cell lines were used to define genomic subclasses of the diseases and identify cell lines representative of each genomic subtype. The algorithm was compared with several traditional clustering methods and showed improved performance. To validate our genomic taxonomy of NSCLC, we correlated the genomic classification with disease outcomes. Overall survival time and time to recurrence were shown to differ significantly between the genomic subtypes.

**Conclusions:**

We developed an algorithm for cancer classification based on genome-wide patterns of copy number aberrations and demonstrated its superiority to existing clustering methods. The algorithm was applied to define genomic subgroups of three cancer types and identify cell lines representative of these subgroups. Our data enabled the assembly of representative cell line panels for testing drug candidates.

## Background

Cancer is a disease of the genome that is characterized by substantial variability in the clinical course, outcome, and response to therapies. A key factor underlying this variability is the genomic heterogeneity of human tumors: individual tumors of the same histopathological subtype and anatomical origin typically carry different aberrations in their cellular DNA. Many of the most efficacious recent drugs target specific genetic aberrations rather than histological disease subtypes, for example trastuzumab and lapatinib for treating HER2-positive breast cancers [1], tamoxifen for treating ER-positive breast cancers[2,3], and gefitinib and erlotinib for non-small cell lung cancer with EGFR mutations [4-8].

Several subtypes of common cancers have been identified based on the aberrations of individual cancer genes, for example HER2-amplified breast cancer [1,9,10], EGFR-mutated and EGFR-amplified non-small-cell lung cancer [5,8], and others. However, cancer is a complex disease driven by the interaction of multiple genes and pathways [11,12]. Therefore, the copy number status of individual genes may not be sufficient to define cancer subtypes and predict the response to treatments. More comprehensive cancer taxonomy needs to be designed based on genome-wide patterns of DNA copy number abnormalities.

Previous ground-breaking studies have reported molecular classifications for key cancer types based on their global patterns of gene expression [13-16]. As the high-density array technology became a reliable tool for copy number profiling, multiple gene copy number datasets were generated, revealing the genomic heterogeneity of key cancer types at the gene copy number level [17]. Various clustering methodologies have been applied to comparative genomic hybridization (CGH) data sets to classify cancers based on their copy number patterns and identify copy number aberration hotspots [17-23]. Taxonomies based on gene copy number have a number of advantages over gene expression-based classifications. In particular, copy number alterations are stable events, not affected by cell cycle or cytokine stimulation. Additionally, they show greater consistency between primary human tumors and cultured cell lines.

Here we developed a copy number-based methodology for cancer classification in order to enable identification of genomic subgroups of major cancer types and facilitate rational selection of tumor models representative of individual subgroups. The methodology is based on the previously published genomic non-negative matrix factorization (gNMF) algorithm [23-26], with several major modifications to enhance the performance. We applied the algorithm to three major tumor types: non-small cell lung carcinoma (NSCLC), colorectal carcinoma (CRC), and malignant melanoma, identified distinct genomic subtypes for each cancer, and identified cell lines representative of each subtype. Our data enabled the assembly of representative cell line panels for testing drug candidates.

## Methods

### Development of a tumor classification methodology based on genome-wide copy number profiles

**Figure 1:**
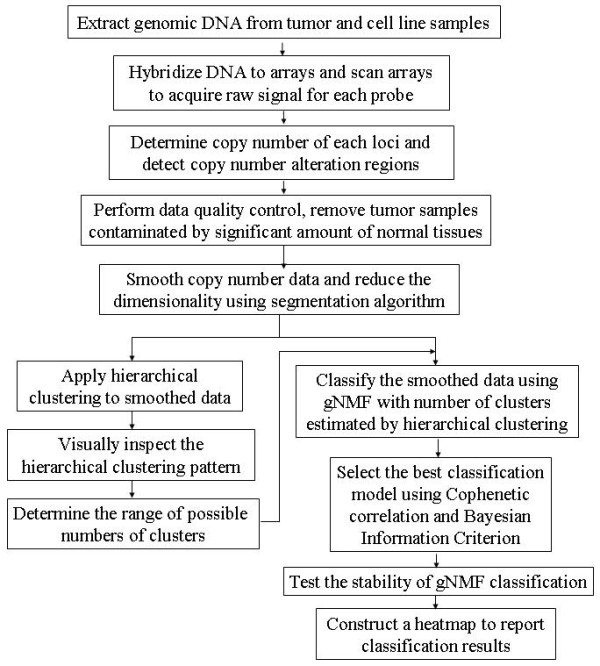
**Workflow of the genomics-based tumor classification procedure**.

The overall flow of our tumor classification methodology is illustrated in Fig. 1. After data pre-processing, a sample quality control procedure was applied to eliminate contaminated samples. For the remaining samples, a pilot hierarchical clustering was first applied to the segment smoothed tumor and cell line CGH data to determine the range of possible numbers of clusters, because the number of clusters needs to be fed into the gNMF algorithm, but is usually unknown for a given data set. The modified gNMF algorithm was then applied to the same set of segment smoothed CGH data to classify it into the initial numbers of clusters suggested by the hierarchical clustering. Using divergence as a stopping criterion and averaging results over multiple initiations, this modification significantly improved the accuracy of clustering at the cost of a higher computational complexity.

To determine the best of the models built by gNMF algorithm with different numbers of clusters, we calculated the Cophenetic correlation coefficient and Bayesian Information Criterion (BIC) for these models, and then selected the one with the minimum BIC or the greatest decrease of Cophenetic correlation. In our study, the minimum BIC and greatest decrease of Cophenetic correlation often pointed to the same model. Finally, the 10-fold stability test was performed on the selected model. Thus, the iteration procedure converges to the best solution, and the optimal model is identified. The entire methodology was implemented using the R language http://cran.r-project.org/. The source code is available from the authors.

### Copy number profiling and primary data processing

The use of human tumor specimens collected at Rush University was approved by the Rush Institutional Review Board. Additional human tumor specimens were obtained from tissue banks (Caprion Proteomics, Montreal, Canada; ProteoGenex, Culver City, CA; Asterand, Detroit, MI; Genomics Collaborative, Inc, Cambridge, MA; and Ontario Tumor Bank, Toronto, Ontario, Canada), and the use was approved by the vendors. Written informed consent was obtained from all the individuals. Genomic DNA was extracted from tumor samples and cell lines using a DNAeasy kit (Qiagen, Valencia, CA). The DNA samples were then processed and hybridized to Affymetrix GeneChip Mapping arrays (Affymetrix, Santa Clara, CA, http://www.affymetrix.com). The arrays were run according to the manufacturer's protocol and scanned using a GeneChip Scanner 3000 G7 (Affymetrix, Santa Clara, CA). The Affymetrix GeneChip^® ^Operating Software (GCOS) collected and extracted feature data from the scanner.

Partek Genomic Suite software (Partek Inc., St. Louis, Missouri, http://www.partek.com/) was used for low-level processing of the raw data to determine the copy numbers at each locus and define regions of copy number alteration. Copy numbers were calculated by comparing the signal intensities for the DNA samples to those for a reference set of 48 normal female tissue samples. The original array scan files and the calculated copy number data were deposited into the Gene Expression Omnibus database http://www.ncbi.nlm.nih.gov/geo/ with series ID GSE20481. The resulting probe-level copy number data were then segmented, and the copy number alteration regions were detected for each sample. Specifically, probe-level copy numbers were segmented into regions using the following control parameters: (i) a region must contain at least 100 probes, (ii) the p-value comparing the mean copy number of the region versus the adjacent regions must be less than 0.00001, and (iii) the signal/noise ratio of the transition must be greater than 0.1. The copy number alteration regions were detected when the mean copy numbers in these regions were lower than 1.65 (deletion) or higher than 2.65 (gain), with P values below 0.01.

### Data quality control

Tumor samples may contain a significant percentage of normal adjacent tissue, which will dilute the signal of copy number alteration and result in false negatives. Therefore, we developed a machine learning algorithm to distinguish between copy number patterns of tumor and normal samples and applied it to identify and eliminate samples contaminated with normal tissue from further analyses. We first selected a subset of samples with the highest number of copy number alteration regions and a set of normal samples. These two groups of samples were used as a training set to train a Random Forest (RF) [27] classifier to classify tumors from normal samples. The trained classifier was then applied to all incoming tumor samples to assign a score to each of them representing the probability of being contaminated by normal tissue. Samples with normal contamination probability over 50% were excluded from our clustering analysis.

### Pilot hierarchical clustering to determine the possible number of subgroups

Although broadly used for clustering of gene expression and copy number patterns, hierarchical clustering is highly sensitive to the distance metrics and typically requires subjective evaluation to define clusters [26]. Nevertheless, this method provides an easy and intuitive way to visualize the overall pattern of the data. The gNMF algorithm requires the number of clusters as input parameter, which is usually unknown for a given data set. In previous applications of gNMF to cluster CGH data, several numbers of clusters were tested, but without an intuitive method to determine these initial numbers [17,23]. We used hierarchical clustering as a pilot to estimate the range of the possible numbers of clusters that exist in the data before applying the gNMF algorithm.

For each data set, we hierarchically clustered the segmented CGH data using Pearson dissimilarity (defined as 1 minus Pearson correlation coefficient). The hierarchical clustering patterns were plotted and visually inspected to derive a range of possible numbers of subgroups in the dataset. These numbers were then used as input in the gNMF clustering analysis.

### The modified genomic non-negative matrix factorization (gNMF) clustering algorithm

Non-negative matrix factorization (NMF) was first suggested by Paatero et al [28]. Like principal component analysis (PCA) or independent component analysis (ICA, [18]), NMF is a linear decomposition algorithm which decomposes a data matrix into a factor matrix and a weight matrix with limited dimensions. However, NMF adds a unique constraint that the factor matrix and weight matrix have only non-negative entries. This feature makes NMF particularly useful in the analysis of positive-value data, such as images, gene expression levels or gene copy numbers. This mathematical tool was further developed by Lee et al. and applied in image analyses [24,25], and then in the analysis of gene expression data [26] and genomic copy number data [17,23].

Given an *n *× *m *matrix *V *of smoothed copy number data for a set of samples, where *n *is the number of segments and *m *is the number of samples, the NMF algorithm factorizes the matrix *V *into an *n *× *r *matrix *W *and a *r *× *m *matrix *H*:

Here *W *can be viewed as the standard model for each subgroup; *H *as the relative weights of each sample belonging to each subgroup; *e *represents the model fitting residues, and *r *is the dimension of decomposition (usually much smaller than *m*). Given *r *and *V *as input, the gNMF algorithm first randomly sets the initial value of *W *and *H*, and then iteratively updates *W *and *H *using the multiplicative update rules:

Where *α *runs from 1 to *r*, *μ *runs from 1 to *m*, and *i *runs from 1 to *n*.

Because of the positive constraint of the NMF algorithm, the result can easily be adapted for clustering purposes by assigning each sample into the cluster (component) that has the maximum weight. Alternatively, under a multiple-run scheme (discussed below), the average correlation of *H *matrix of multiple runs can be used to assign samples into clusters. Therefore, gNMF can also serve as a tool for unsupervised clustering.

Here we introduced several modifications to the gNMF algorithm. In previous applications of gNMF to cluster CGH data, the algorithm was stopped when the subgroup assignments of samples did not change after a pre-defined number of steps (e.g. 100) [17,23]. Based on our tests with simulated data as well as actual CGH data, we determined that this criterion might stop the procedure too early, suggesting that the results could potentially be further improved if the algorithm were allowed to run more steps. Therefore, we modified the algorithm so that after every 100 steps of multiplicative updating the divergence [25] of the current model from the data was calculated, and the iterative algorithm was stopped if the divergence did not decrease by more than 0.001% of the previous divergence calculated 100 steps earlier.

Since gNMF is a stochastic procedure, the algorithm may generate different outcomes when started from different initial values. To further improve the performance of gNMF, we implemented a new multiple initiation strategy. For each data set, we ran the gNMF algorithm 200 times following the above stop criterion, calculated the Pearson correlation coefficient matrix of *H *from the output of each of the 200 random gNMF runs, and averaged the correlation matrices over the 200 runs. The final clustering result was derived by running a hierarchical clustering algorithm using one minus the average correlation matrix as the distance matrix and cutting the dendrogram into the given number of subgroups.

### Model selection using Bayesian Information Criterion and Cophenetic correlation

The core gNMF algorithm was run with different numbers of clusters, *r*, as suggested by the pilot hierarchical clustering. After that, to select the best model, we calculated the Cophenetic correlation coefficient and Bayesian information criterion (BIC) for these models, and then selected the one with the minimum BIC or the greatest decrease in Cophenetic correlation.

The Cophenetic correlation coefficient [29] is a measure of how faithfully a dendrogram that is used to derive the final clustering result preserves the pairwise distances between the original unmodeled data points. Suppose the original data *X*_*i *_have been modeled by a dendrogram *T*_*i*_. Distance measures are defined as follows:

*x*(*i*, *j*) = |*X*_*i *_- *X*_*j*_|, the distance between the *i*^th ^and *j*^th ^samples.

*t*(*i*, *j*) = the dendrogrammatic distance between the model points *T*_*i *_and *T*_*j*_. This distance is the height of the node at which these two points are first joined together.

Then, if *x *is the average of *x*(*i*, *j*), and *t *is the average of *t*(*i*, *j*), the Cophenetic correlation coefficient *c *is defined as follows:

The Cophenetic correlation coefficient has been used to select the best model built by the gNMF algorithm in previous applications [17,23], and it has been reported that with the increase of *r*, the Cophenetic correlation will decrease dramatically at certain point which suggests the best number of clusters.

The Bayesian Information Criterion (BIC) [30] is also widely used in statistical applications for model selection purposes. BIC is defined as follows:

where *L *is the likelihood which measures how good the model approximate the data, *k *is the number of parameters used in the model, and *n *is the number of samples. The second term, *k*ln*(n)*, serves as a penalty on the number of parameters used in the model to avoid overfitting. A lower BIC value usually represents a good model with relatively higher likelihood and also relatively lower risk of overfitting.

Lognormal distribution is widely used to fit DNA copy numbers [31]. To calculate the likelihood, we assume samples in each cluster come from the same multi-lognormal distribution where the mean copy number of each segment follows a lognormal distribution. The correlation between segments was weak, so independence was assumed between segments in the calculation. The resulting log-likelihood was

where *r *is the number of clusters, *n*_*i *_is the number of samples in cluster *i*, and *m *is the number of segments, *y*_*ijt *_is the log transformed copy number of the *t*^th ^segment of the *j*^th ^sample in the *i*^th ^cluster, *μ*_*it *_is the average of log transformed copy numbers of the *t*^th ^segment in the *i*^th ^cluster, and *σ*_*it *_is the standard deviation of log transformed copy numbers of the *t*^th ^segment in the *i*^th ^cluster. Then the number of parameters, *k*, in the specified model would be 2 × *r *× *m*.

In our studies, the minimum BIC and greatest decrease of Cophenetic correlation often pointed to the same model.

### 10-fold cross-validation test of clustering stability

A 10-fold cross-validation test was used to assess the stability of clustering results. After assigning samples into subgroups, we randomly left 10% of samples out and applied the same procedure to the remaining 90% of samples using the same control parameters. The number of samples that were assigned to a different subgroup was counted as errors. This test was repeated 200 times to derive an error rate, which represented the stability of the clustering result with respect to the permutation of samples.

## Results

### Classification of NSCLC tumors and cell lines

**Figure 2:**
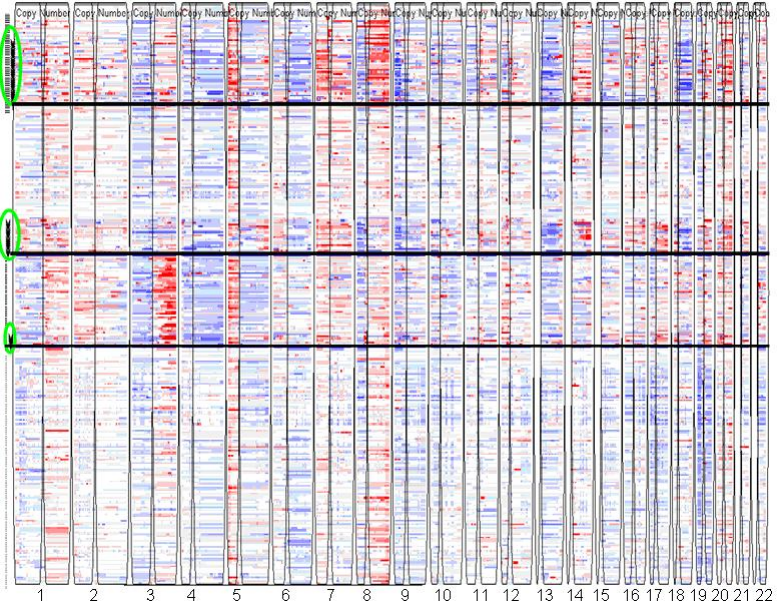
**Heatmap of the NSCLC tumor and cell line CGH data classified into 4 clusters**. Each row represents a sample and each column represents a SNP locus. Red, white, and blue colors indicate high, normal, and low copy numbers, respectively. Horizontal black lines separate different subgroups, and vertical spaces separate chromosome 1 to 22. Cell lines representing each cluster are highlighted by green circles.

We applied the gNMF-based classification methodology above to a dataset for 245 NSCLC tumors and 57 NSCLC cell lines that contained both internally generated and public Affymetrix Human GeneChip^® ^Mapping 100K array data [32]. The Affymetrix 100K array measures the copy number of > 100,000 Single-nucleotide polymorphism (SNP) loci covering the entire human genome with an average distance between SNPs of 23.6 kb. The initial processing yielded 11,419 copy number segments, and no sample was detected to be contaminated by normal tissue. Dimension reduction resulted in 8,172 segments for the 302 samples. Pilot hierarchical clustering of the segments (Additional file 1**Fig. S1**) suggested the existence of 3-8 major clusters in the data. The 302 tumors and cell lines were then clustered using the gNMF algorithm with cluster numbers 3-8, and the Cophenetic correlations and BICs for these gNMF models were calculated (Table 1). The model with four clusters had the minimum BIC, and between four and five clusters the Cophenetic correlation had the greatest decrease. Therefore, the four-cluster model was chosen for the NSCLC data set. The heatmap of the four-cluster model is shown in Fig. 2. The cell lines selected to represent each subtype of NSCLC are listed in Table 2. They can serve as disease models for the respective subtypes of NSCLC.

**Table 1:** Cophenetic correlation and BIC of the NSCLC gNMF models under different cluster numbers.

***r***^***a***^	*Cophenetic correlatoin*	*BIC*
3	0.8031	1032670
4	0.7664	992443
5	0.7103	1249580
6	0.7166	1301055
7	0.7040	1301808
8	0.7109	1202876

^a^. The number of clusters by gNMF.

**Table 2:** Number of tumor patients in each of the four NSCLC subtypes and the cell lines selected to represent each subtype.

Clusters	Number of tumors	Representative Cancer Cell Lines for each subtype
Cluster A	19	HCC827, NCI-H1437, NCI-H1563, NCI-H1568, NCI-H1623, NCI-H1651, NCI-H1693, NCI-H1755, NCI-H1793, NCI-H1838, NCI-H1944, NCI-H1975, NCI-H1993, NCI-H2023, NCI-H2073, NCI-H2085, NCI-H2087, NCI-H2122, NCI-H2126, NCI-H2228, NCI-H2291, NCI-H23, NCI-H2342, NCI-H2347, NCI-H647, NCI-H920, NCI-H969, CLS-54, LX-289, SK-LU-1, H2882, Calu-6, H358, H460

Cluster B	60	NCI-H2405, NCI-H522, SK-MES-1, H157, H1819, H2009, H2887, HCC1171, HCC1359, HCC15, HCC193, HCC366, HCC461, HCC515, HCC78, HOP-62, HOP-92, NCI-H266

Cluster C	42	A549, Calu-3, NCI-H1734, NCI-H838, HCC95

Cluster D	124	

### Biological significance of the NSCLC classification

Thus, we identified genomic subgroups of NSCLC using the unsupervised clustering methodology above and selected representative cell lines for each subtype. However, the biological significance of the subgroups of NSCLC was still unclear. One way to determine the phenotypical relevance of a classification is to test the difference in the clinical outcome of patients assigned into the subgroups.

**Figure 3:**
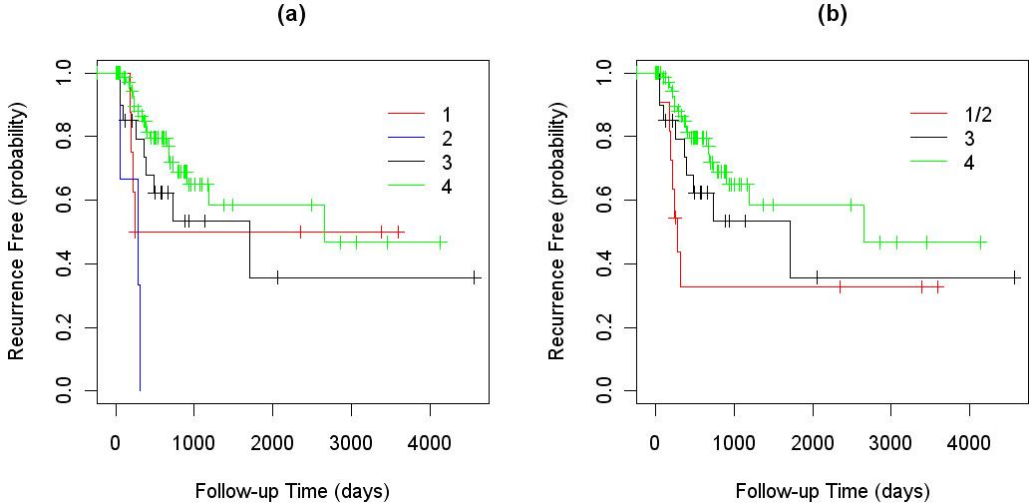
**Kaplan-Meier curves of the TTR for clinically annotated samples in the four NSCLC clusters: (a) four clusters considered separately; and (b) clusters 1 and 2 combined**.

Among the 245 NSCLC tumor samples used to identify subtypes of NSCLC, clinical outcome information (overall survival: OS; time to recurrence: TTR) was available for a subset of 111 samples. The numbers of outcome-annotated samples in clusters 1, 2, 3, and 4 were 9, 3, 21 and 78, respectively. The logrank test showed that the TTRs of the four clusters were significantly different with a P-value of 0.001. The median TTRs for the four groups were 1918, 281, 1710 and 2657 days. Since there were only three clinically annotated samples in cluster 2, we re-ran this analysis after combining the samples in clusters 1 and 2. The combined samples also had a significantly lower TTR than those in the other two clusters (P-value 0.040), with the median TTR of 281 days. The Kaplan-Meier curves for these analyses are shown in Fig. 3.

To further validate the ability of our algorithm to classify tumors into biologically relevant genomic groups, an independent set of 71 NSCLC tumor samples was profiled for copy number alterations, and the data were segmented. These validation samples were then matched to the four NSCLC clusters.

**Figure 4:**
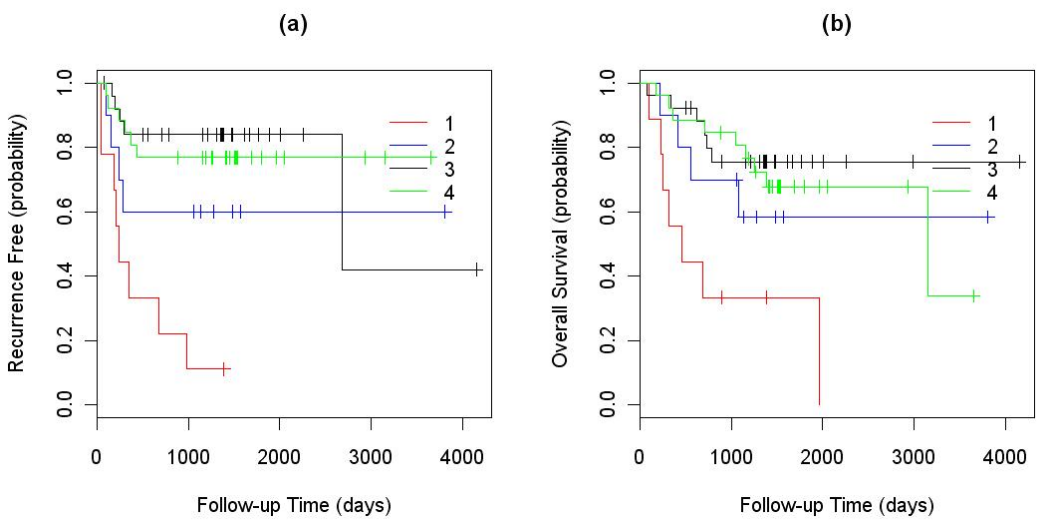
**Kaplan-Meier curves of the TTR and OS between the validation samples assigned into the four clusters using all existing tumor and cell lines to represent the clusters: (a) TTR; and (b) OS**.

First, all tumors and cell lines in our defined clusters were used to represent the clusters. We calculated Pearson correlation coefficients between the validation samples and each of the cell lines and tumors in the four clusters. The validation samples were then assigned to the cluster that contained the best matched representative cell line or tumor based on the correlation coefficients. Finally, the differences in TTR and OS of the validation samples assigned into different clusters were analyzed using a logrank test, and their Kaplan-Meier curves were plotted (Fig. 4). In this analysis, both TTR and OS showed significant differences between the four clusters with P-values of 4.7E-5 and 0.002, respectively. The median TTRs of the validation samples assigned to the first and third clusters were 246 and 2679 days, and that for second and forth clusters were not reached (less than 50% of the patients in the second and forth clusters have recurrence in the study). The median OS value of the validation samples assigned to the first and fourth clusters were 464 and 3147 days, and that for the second and third clusters were not reached. Samples assigned to cluster 1 had a significantly lower TTR and OS than samples assigned to other clusters (Fig. 4).

**Figure 5:**
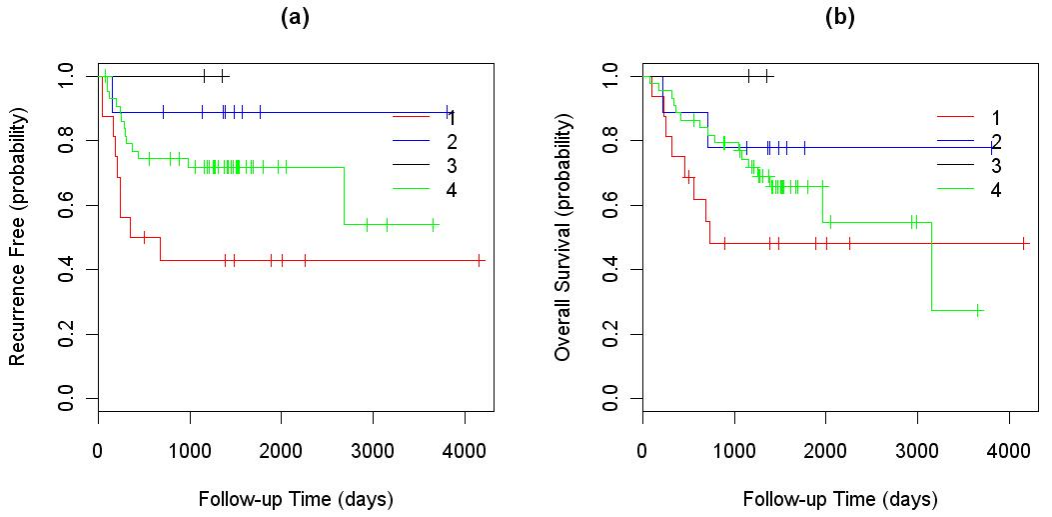
**Kaplan-Meier curves of the TTR and OS between the validation samples assigned into the four clusters using cell lines to represent the first 3 clusters and tumors to represent the 4th cluster: (a) TTR; and (b) OS**.

Additionally, to test the cell line panel assembled, we used only cell lines to represent the first three clusters, while assigning the validation samples into clusters using the highest Pearson correlation coefficient criterion. Since the fourth cluster did not contain any cell lines, all tumor samples in the cluster were used to calculate Pearson correlation coefficients and assign validation samples. In this analysis, the difference in TTRs between the four clusters was still significant (P-value 0.045) with the median TTR of 512 days for validation samples assigned to cluster 1 but not reached for the remaining clusters (Fig. 5a). The difference in OS between the four clusters was not significant (P-value 0.25) for the validation samples, but the Kaplan-Meier curve showed a trend for lower OS for samples in cluster 1 relative to the other clusters (Fig. 5b) with the median OS of 733, not reached, not reached and 3147 days for the 4 clusters. When samples in clusters 2, 3, and 4 were combined and compared to samples in cluster 1, the P-value was marginally significant (P-value = 0.116, Kaplan-Meier curve not shown) with the median OS of 733 vs. 3147 days.

Thus, the NSCLC clusters identified using our clustering methodology are associated with different disease outcomes, suggesting that these genomic clusters represent clinically distinct subgroups of the disease. Consequently, the cell lines selected can be used as models to represent the existing subtypes of the disease.

### Comparison with other unsupervised clustering algorithms

We used the NSCLC dataset to compare the performance of our method with several known unsupervised clustering methodologies, namely the hierarchical clustering, the original gNMF method [17,23-26], and K-means clustering [33]. The same NSCLC dataset was clustered into 4 subgroups by these methods. The ability of the procedures to generate clinically relevant tumor subgroups was used as the main performance criterion. The cluster stability was assessed as an additional performance metric.

The performance comparison of the various clustering methods is presented in Table 3. In the TTR analysis, the p-values for Hierarchical Clustering, the original gNMF, and K-means clustering were 0.168, 0.085, and 0.566, respectively. In the OS analysis, the p-values were 0.137, 0.144 and 0.413, respectively. Thus, the clustering patterns generated by these procedures did not correlate significantly with the clinical outcomes. Whereas our method yielded a significant P value of 0.001 for separating NSCLC tumor patients with different TTR, implying that it classifies samples into biologically relevant subgroups.

**Table 3:** Performance comparison of the unsupervised clustering algorithms on NSCLC data set.

Methods	**P-value for TTR**^**a**^	**P-value for OS**^**b**^	**Stability Test**^**c**^
our gNMF	0.001	0.250	14.24%

hierarchical clustering	0.168	0.137	23.32%

original gNMF	0.085	0.144	21.59%

K-means	0.566	0.413	23.27%

^a ^The p-values comparing TTR of NSCLC patients classified into different subtypes by each clustering method

^b ^The p-values comparing OS of NSCLC patients classified into different subtypes by each clustering method

^c ^The error rate of 10-fold stability test for each method

To evaluate the relative stability of clusters produced by different procedures, the 10-fold cross-validation scheme was applied. For our algorithm, the total error rate for the NSCLC data set was 14.24%, while the error rates for hierarchical clustering, the original gNMF, and K-means clustering were 23.32%, 21.59%, and 23.27%, respectively. Thus, our comparison shows that the tumor classification methodology described here outperforms the existing algorithms, both in terms of cluster stability and the biological relevance of clusters.

### Classification of colorectal and melanoma tumors and cell lines

**Figure 6:**
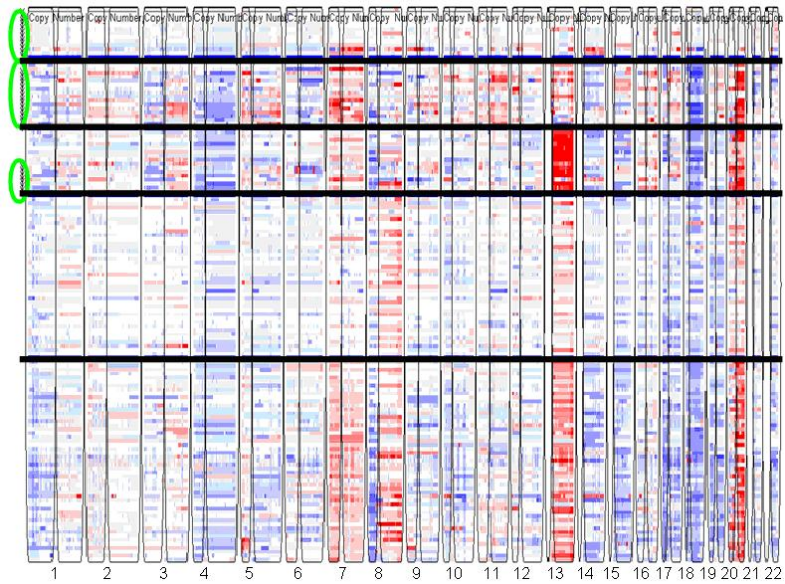
**Heatmap of the CRC tumors and cell lines classified into 5 clusters**.

**Figure 7:**
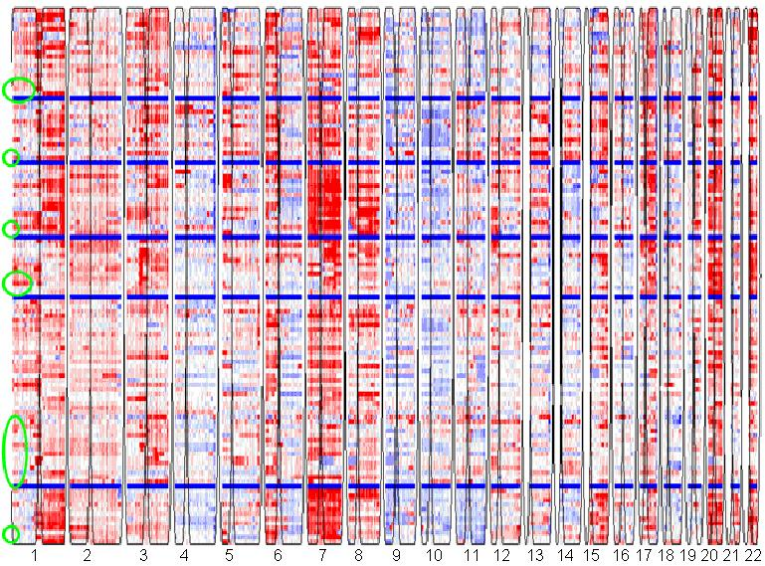
**Heatmap of the melanoma tumors and cell lines classified into 6 clusters**.

Our classification methodology was also applied to two additional copy number datasets, one for colorectal cancer (CRC) and the other one for malignant melanoma. Normal contaminated samples were detected and removed before clustering. Pilot hierarchical clustering was used to determine the initial numbers of clusters (Additional file 1**Figs. S2, S3**), then the gNMF clustering algorithm was applied. In the CRC study, 101 tumors and 35 cell lines were classified into five distinct genomic clusters. The heatmap is shown in Fig. 6, and the cell lines selected to represent each subtype are listed in Table 4. In the melanoma study, 30 cell lines and 80 short-term melanoma cultures were clustered into six distinct genomic clusters. The heatmap is shown in Fig. 7, and the cell lines selected to represent each subtype are listed in Table 5. These cell lines can serve as disease models for the respective subtypes of CRC and melanoma. Clinical information for these tumors was not available, so we were not able to evaluate the biological significance of these clusters. However, we believe that the genomics-based cell line panels for these cancer types will improve screening of drug candidates by ensuring representation of all genomic subtypes.

**Table 4:** Number of tumor patients in each of the five CRC subtypes and the cell lines selected to represent each subtype.

Clusters	Number of tumors	Representative Cancer Cell Lines for each subtype
Cluster A	0	HCT-8, LS 174T, SK-CO-1, SW48, DLD-1, HCT-15, HCT116, LoVo, CL-34, CL-40, C170, LS180

Cluster B	2	Caco-2, LS1034, LS411N, LS513, NCI-H498, NCI-H747, SW1116, SW1417, SW837, HT-29, SW620, CL-11, CL-14, Colo-678, SW-480

Cluster C	8	Colo 320DM, NCI-H508, NCI-H716, SW1463, SW403, SW948, Colo 205, Colo-206F

Cluster D	40	

Cluster E	51	

**Table 5:** Number of tumor patients in each of the six melanoma subtypes and the cell lines selected to represent each subtype.

Clusters	Number of tumors	Representative Cancer Cell Lines for each subtype
Cluster A	15	SKMEL119, HS944, WM1366, WM88

Cluster B	12	WM3248

Cluster C	14	1205LU

Cluster D	4	451LU, SKMEL19, SKMEL28, SKMEL30, SKMEL63, WM35, WM983, WM983C

Cluster E	25	WM3211, M14, MEWO, SKMEL2, SKMEL5, UACC257, UACC62, WM122, WM13662, WM239A, WM32112, WM32482, WM793B, 501MEL

Cluster F	10	MALME3M, WM882

## Discussion

Currently, pre-clinical models for oncology drug testing are selected based on their availability, adaptability to tumor formation in mice, growth in culture, as well as other parameters. This approach does not take into account the genetic heterogeneity of the parent tumor, resulting in poor representation of molecular subtypes of tumors during preclinical testing. Thus, the high response rates that are frequently seen in pre-clinical testing may only represent the response of the single molecular subtype represented in the preclinical testing laboratory. If this subtype represents only a fraction of the patient population, and if the drug is efficacious only against this specific subtype, then the response rate in the clinic will be significantly lower. Therefore, there is a need for improved pre-clinical testing models that would cover a broader spectrum of parent tumors. Such improved pre-clinical testing will increase the predictability of the preclinical testing of new drugs.

Recently, application of high-density SNP genotyping microarrays for gene copy number profiling enabled generation of comprehensive copy number datasets for most major tumor types. However, the processing and use of high-density copy number for cancer classification remains a challenge due to the complexity of using alterations of varied length and amplitude in clustering samples. We believe that copy number profiles are best suited for developing a genomic taxonomy of cancer due to their temporal stability and easier detection in various samples. Therefore, we developed an unsupervised methodology based on revised gNMF algorithm for classifying samples based on their genomic patterns of copy number aberrations and applied it to three datasets, which contained both internally generated and public data. The NMF algorithm has previously been used in image analysis [24,25]. It has been adapted for use in gene expression profiling [26] and gene copy number analysis [17,23]. It has shown advantages over traditional clustering and component analysis algorithms such as Princial Component Analysis (PCA), Self-Organizing Maps (SOM), and hierarchical clustering when applied to gene expression data [26]. We further improved the original gNMF algorithm and developed an integrated workflow, which includes pilot hierarchical clustering to estimate the possible number of clusters, gNMF with a revised stop criterion and multi-run strategy, model selection using Cophenetic correlation and BIC, and a cross-validation stability test.

We applied our algorithm to datasets for NSCLC, CRC, and melanoma, each containing copy number profiles for hundreds of patient tumors and ~50 cell lines. The algorithm identified distinct new genomic subtypes for each tumor type. The clustering results were then evaluated for stability and reproducibility by using 10-fold cross-validation schemes. To determine whether the clusters discovered have biologically meaningful differences, we correlated the available disease outcome parameters (overall survival and time to recurrence) with the cluster distribution of samples and found a significant difference in clinical outcome between samples assigned into different clusters. As an additional test to validate the correlation between the classification and the outcome, we also profiled a group of outcome-annotated validation samples and assigned them to the existing clusters based on their copy number profiles. It was shown that the cluster assignment of the samples also significantly correlates with the clinical outcome, providing additional validation to our methodology.

Copy number alterations of single oncogenes and tumor-suppressor genes have previously been implicated as important biomarkers in cancer. Notable examples include HER2 amplification in breast cancer [1,9,10], EGFR amplification in lung cancer [4-8], and MYCN amplification in neuroblastoma [34,35]. In these and other examples of previous validation and use of copy number alterations as predictors of outcome and response to therapeutic agents, only individual alterations were considered. Our methodology is based on the analysis of complex whole-genome wide patterns of copy number alterations. Therefore, it provides a complete characterization of genomic subtypes of the cancer under consideration and is expected to generate more precise correlates of clinical behaviour and response to drugs. We believe that the proposed genomic taxonomy is valid for the entire population of tumor patients, because (i) the sample sets used to develop it were large enough (150~300 samples) and (ii) the samples were acquired from a variety of sources, thus eliminating the possibility of bias.

Heatmaps of our clustering models shown in Figs. 2, 6, and 7 revealed major genomic aberrations characteristic of individual clusters, such as 8q gain for cluster 1 or 3q gain for cluster 3 of NSCLC, 13q gain for cluster 3 or 18q loss and 20q gain for cluster 2 of CRC, and chr7 gain for cluster 3 of melanoma. However, we would like to emphasize the fact that the classification patterns observed are driven by the genome-wide copy number aberration profiles rather than selected major aberrations. In many cases, smaller copy number aberrations contain important cancer genes. Some aberrations may also represent secondary events that are part of the tumor's genomic signature. To identify the exact genes that differentiate between clusters in a statistically rigorous manner, we would need to address several complex issues related to variable selection from high-dimensional data, including controlling for false-positives and false-negatives, correlation between variables, and the lack of reproducibility between studies due to systematic biases. Analysis of these issues is beyond the scope of this manuscript.

We are currently testing the classification methodology for its ability to predict disease outcome for new patients. To predict the disease outcome for a new patient, a tumor sample will be profiled for copy number alterations by high-density arrays and assigned into one of the subgroups. Alternatively, a panel of FISH probes may be designed based on the most characteristic copy number abnormalities for each subgroup, and new patient samples would be tested by FISH using the probe panel developed and classified into one of the subgroups based on the pattern of aberrations observed. The association with one of the groups would be used to predict response to the agent under consideration. A companion diagnostic can thus be developed for use with the drug considered. An additional application of the proposed classification methodology is in the assembly of a collection of preclinical testing models representative of the genetic diversity of tumors, such as the NSCLC, CRC, and melanoma testing panels that are described here.

## Conclusion

We developed a modified genomic non-Negative Matrix Factorization (gNMF) clustering algorithm to cluster CGH data of tumor and cell lines to identify genomic subtypes of tumors and select representative cell lines. Our algorithm enables the assembly of panels of cell lines representative of the genomic heterogeneity of cancers.

## Competing interests

The methodologies developed in this manuscript and the cell panels representing different subtypes of NSCLC, CRC and melanoma have been claimed in US Patent Applications No. 61/110,281, 61/110,317, 61/110,308 and 61/110,296 ( submitted by Abbott Laboratories).

## Authors' contributions

CVS and DS initiated the study and generated internal CGH data used in this study. XL and KZ developed the statistical methodologies. XL wrote the R code for the methodology and carried out the experiments. JC provided part of the NSCLC patient samples with clinical outcome, and provided guidance on using clinical information to validate the clustering results. DS managed the entire study. XL and DS wrote the manuscript with input from all authors. All authors read and approved the final manuscript.

## Pre-publication history

The pre-publication history for this paper can be accessed here:

http://www.biomedcentral.com/1755-8794/3/23/prepub

## Supplementary Material

Additional file 1 Additional figures for the gNMF based unsupervised clustering algorithm. Click here for file
