# Supplementary material for: An algorithm for classifying tumors based on genomic aberrations and selecting representative tumor models
Source: BMC Med Genomics. 2010 Jun 22;3:23. doi: 10.1186/1755-8794-3-23 (PMC2901344; doi:10.1186/1755-8794-3-23)
Supplement: Additional file 1 — Additional figures for the gNMF based unsupervised clustering algorithm. [file 1755-8794-3-23-S1.DOC]

**Additional file for**

# An algorithm for classifying tumors based on genomic aberrations and selecting representative tumor models

### Xin Lu^*1^, Ke Zhang^2^, Charles Van Sant^3^, John Coon^4^, and Dimitri Semizarov^*1^

^1^ Global Pharmaceutical Research and Development, Abbott Laboratories, 100 Abbott Park Road, Building AP-10, Dep. R4CD, Abbott Park, IL 60064, USA.

^2^ Current address: Department of Pathology, School of Medicine & Health Sciences, University of North Dakota, 501 N. Columbia Road, Grand Forks, ND 58202, USA.

^3^ Current address: Astellas Pharma Global Development, INC. 8045 Lamon Ave, Skokie, IL 60077, USA.

^4^ Department of Pathology, Rush University Medical Center, Chicago, IL 60612, USA.

* To whom correspondence should be addressed.

**Additional figures**


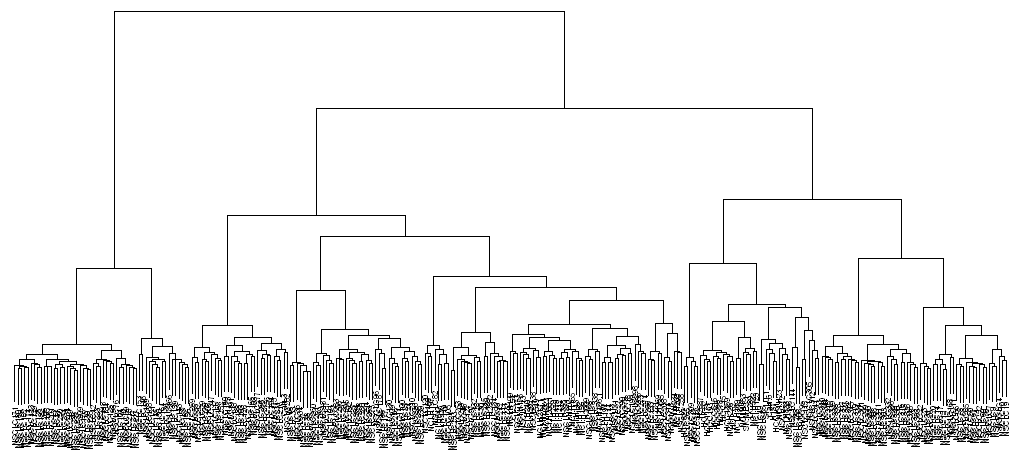


**Figure S1**. Pilot hierarchical clustering of the NSCLC data set.


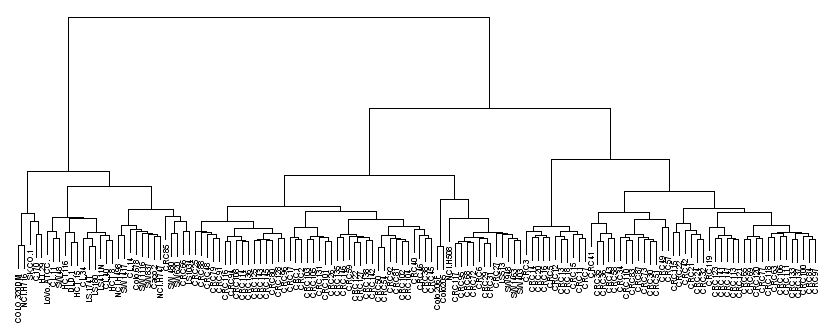


**Figure S2**. Pilot hierarchical clustering of the CRC data set.

**
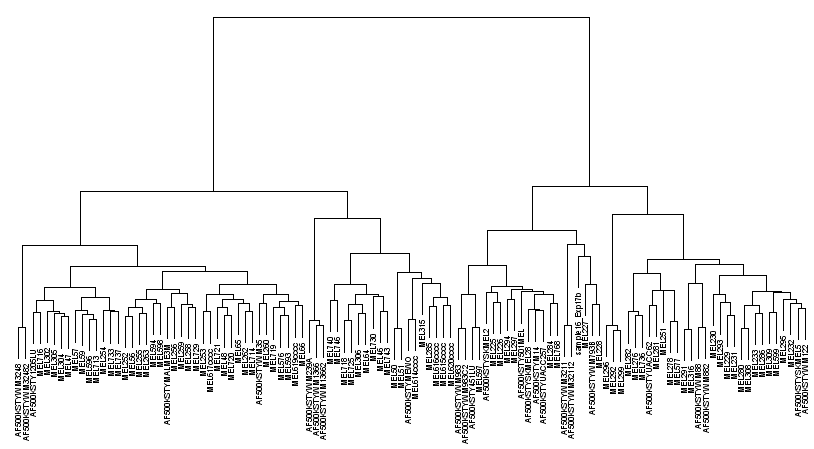
**

**Figure S3**. Pilot hierarchical clustering of the malignant melanoma data set.
